# Molecular Characterization of the Bacterial Community in Biofilms for Degradation of Poly(3-Hydroxybutyrate-*co*-3-Hydroxyhexanoate) Films in Seawater

**DOI:** 10.1264/jsme2.ME17052

**Published:** 2018-03-29

**Authors:** Tomohiro Morohoshi, Kento Ogata, Tetsuo Okura, Shunsuke Sato

**Affiliations:** 1 Department of Material and Environmental Chemistry, Graduate School of Engineering, Utsunomiya University 7–1–2 Yoto, Utsunomiya, Tochigi 321–8585 Japan; 2 GP Business Development Division, Kaneka Corporation 1–8 Miyamae-Cho, Takasago-Cho, Takasago, Hyogo 676–8688 Japan

**Keywords:** biodegradable plastic, poly(3-hydroxybutyrate-*co*-3-hydroxyhexanoate), biofilm, microbial community, degradation

## Abstract

Microplastics are fragmented pieces of plastic in marine environments, and have become a serious environmental issue. However, the dynamics of the biodegradation of plastic in marine environments have not yet been elucidated in detail. Polyhydroxyalkanoates (PHAs) are biodegradable polymers that are synthesized by a wide range of microorganisms. One of the PHA derivatives, poly(3-hydroxybutyrate-*co*-3-hydroxyhexanoate) (PHBH) has flexible material properties and a low melting temperature. After an incubation in seawater samples, a significant amount of biofilms were observed on the surfaces of PHBH films, and some PHBH films were mostly or partially degraded. In the biofilms that formed on the surfaces of unbroken PHBH films, the most dominant operational taxonomic units (OTUs) showed high similarity with the genus *Glaciecola* in the family *Alteromonadaceae*. On the other hand, the dominant OTUs in the biofilms that formed on the surfaces of broken PHBH films were assigned to the families *Rhodobacteraceae*, *Rhodospirillaceae*, and *Oceanospirillaceae*, and the genus *Glaciecola* mostly disappeared. The bacterial community in the biofilms on PHBH films was assumed to have dynamically changed according to the progression of degradation. Approximately 50 colonies were isolated from the biofilm samples that formed on the PHBH films and their PHBH-degrading activities were assessed. Two out of three PHBH-degrading isolates showed high similarities to *Glaciecola lipolytica* and *Aestuariibacter halophilus* in the family *Alteromonadaceae*. These results suggest that bacterial strains belonging to the family *Alteromonadaceae* function as the principal PHBH-degrading bacteria in these biofilms.

Plastic pollution became a social issue in the 1980s and has had a devastating effect on the environment and wildlife (10). In the marine environment, examinations of encounters according to species revealed that all known species of sea turtle and more than half of all known species of marine mammal and sea bird had ingested or become entangled in marine plastic debris (6). Many species of marine wildlife mistake plastic for prey and ingest it, which causes damage to the digestive tracts of these animals (27). Organic pollutants attached to the surface of plastic have been shown to accumulate pollutants and transport them through ocean currents (17). So-called “microplastics”, which means smaller pieces of plastic mainly fragmented by UV radiation in marine environments, have recently become a serious environmental issue. Microplastics are defined as particles of less than 5 mm in size and various chemicals and toxins are attracted onto their surfaces (2, 3). There are concerns regarding the transport of these compounds from invertebrates to mammals through the food chain. Therefore, the dynamics of the distribution, diffusion, and degradation of microplastics in marine environments are being examined in more detail in an attempt to overcome this issue.

Biodegradable plastics may be broken down by various microorganisms into carbon dioxide (CO_2_) (29). Poly(ɛ-caprolactone) (PCL), polybutylene succinate (PBS), poly(butylene succinate-*co*-butylene adipate) (PBSA), and poly(butylene adipate-*co-*terephthalate) (PBAT) are categorized as oil-based biodegradable plastics and their ester bonds may be degraded by certain enzymes secreted by microorganisms (12, 29). Poly(lactic acid) (PLA) and polyhydroxyalkanoates (PHAs) are categorized as bio-based biodegradable plastics and are derived from renewable resources (29). PHAs are polyesters that are synthesized by a wide range of microorganisms from renewable resources such as sugars, plant oils, and glycerol (1, 8). PHA-degrading bacteria and fungi have been isolated from various environments and produce extracellular PHA depolymerases to degrade PHAs under anaerobic and aerobic conditions (16). Briefly, one of the PHA derivatives, poly(3-hydroxybutyrate) is degraded to 3-hydroxybutyrate (3HB) monomers or oligomers by PHA depolymerase. These oligomers are then hydrolyzed to monomers by 3HB-oligomer hydrolase. 3HB monomers are converted into acetoacetate by 3HB dehydrogenase. Since PHAs have excellent biodegradability, they have been expected to play an important role in the protection of marine environments and ecosystems as well as in the reduction of CO_2_ emissions, a cause of global warming, when substituted with conventional plastic materials (25). One of the PHA derivatives, poly(3-hydroxybutyrate-*co*-3-hydroxyhexanoate) (PHBH) possesses flexible material properties and a low melting temperature, which is effective for lower pyrolysis temperatures. Therefore, PHBH is expected to be applicable as, for example, sheets, films, and fibers (21). Since PHA-degrading bacteria have also been isolated from marine environments (13), plastic products made from PHBH are expected to be degradable in marine environments. Therefore, PHBH is assumed to not significantly contribute to microplastic issues because it will be rapidly degraded in marine environments.

A biofilm is an assembly of surface-associated microbial cells that is enclosed in an extracellular polymeric substance matrix such as polysaccharide (5). Biofilm formation generally exerts harmful effects in natural, clinical, and industrial environments (5). On the other hand, the biofilm formation of plastic-degrading soil isolates on plastic surfaces may be beneficial for the biodegradation of these complexes (7). However, the relationship between biofilm formation and the biodegradation of plastic in marine environments has not yet been elucidated. In the present study, we investigated biofilm formation and the biodegradation of general biodegradable plastic films in seawater, and reported a relationship between biodegradability and biofilm formation on the surfaces of several plastic films and PHBH films.

## Materials and Methods

### Biofilm formation on plastic film surfaces

Seawater samples were collected at 34°44.37′ N/134°47.27′ E, known as Takasago harbor, on March 15, April 19, and June 8, 2016. In order to assess biodegradability in seawater, six types of general biodegradable plastic films: PLA, PBAT, PBS, PBSA, PCL, and PHBH, were used. These plastic films have been described in Table 1. All films had a thickness of approximately 100 μm, and were prepared by T die cast extrusion. Seawater samples were mixed with 0.5 g L^−1^ NH_4_Cl as the N source and 0.1 g L^−1^ KH_2_PO_4_ as the P source. Rectangular plastic films with dimensions of 2×3 cm were cut from plastic sheets and hung down in 100-mL screw-cap glass bottles. One hundred milliliters of seawater samples were poured into the screw-cap glass bottles and incubated at 23°C with gentle shaking at 150 rpm until the formation of biofilms or breakdown.

### Construction and sequencing of the 16S rRNA clone library

Plastic films were transferred into 50-mL centrifuge tubes and 5 mL of sterile water was added. Biofilms were unglued from the surfaces of plastic films by vortexing and collected by centrifugation at 10,000×*g* for 5 min. Total DNA was extracted from enriched biofilm samples by the DNeasy Blood & Tissue Kit (Qiagen, Tokyo, Japan). The 16S rRNA genes were amplified from total DNA by PCR with Blend Taq-Plus DNA polymerase (Toyobo, Osaka, Japan) and the previously described primers, 27f (5′-AGAGTTTGATCMTGGCT CAG-3′) and 1525r (5′-AGGAGGTGWTCCARCC-3′) (20). PCR was performed using the following cycling parameters: 94°C for 30 s, 50°C for 30 s, and 74°C for 1 min for 27 cycles. PCR products were cloned into a pGEM-T easy vector (Promega, Tokyo, Japan). The clone library sequencing of 16S rRNA genes was performed by the Takara Bio Biomedical Center (Takara Bio, Shiga, Japan). A partial sequence of the 16S rRNA gene was obtained using the 27f primer. Sequences were manually edited to eliminate primer sequences and low-quality regions. Approximately 600 bases of the 16S rRNA gene were then used for sequence analyses.

### Clustering of 16S rRNA sequences

The clustering of 16S rRNA for operational taxonomic unit (OTU) prediction was performed according to a previously described method with slight modifications (24). Briefly, 16S rRNA sequences were aligned using the ClustalW program (28). Based on this alignment, a distance matrix was constructed using the DNADIST program from PHYLIP software ver. 3.69 (http://evolution.genetics.washington.edu/phylip.html) with default parameters. The resulting matrices were run in the Mothur program (22) to generate diversity indexes and clustering analyses. OTUs were defined with ≥97% identity for clustering analyses. The phylogenetic tree was constructed using the neighbor-joining method with the ClustalW program of MEGA7 (http://www.megasoftware.net/).

### Screening for PHBH-degrading bacteria from biofilms

Suspensions of biofilms were serially diluted with distilled water, spread on Marine broth 2216 (MB; Difco, Tokyo, Japan) agar plates, and incubated at 30°C for 72 h. Colonies from each sample were randomly selected and transferred onto fresh MB agar medium. In order to detect PHBH-degrading activity, an MB agar plate containing PHBH (MB-PHBH) was prepared. Briefly, PHBH powder (Kaneka Corporation, Hyogo, Japan) was mixed with MB medium at a concentration of 1 g L^−1^ and sonicated to obtain better dispersion. After autoclaving, 10 mL of agar medium was poured into 60-mm plastic Petri dishes. Bacterial colonies were streaked on the center of the MB-PHBH plates. After an incubation at 30°C for 3 d, the development of clear zones around the colonies was evaluated as the degradation of PHBH. Biofilm formation on PHBH films by PHBH-degrading isolates was evaluated using a liquid medium assay. Bacterial isolates were grown in MB medium at 30°C for 15 h. Full-grown cultures were inoculated (a 1% inoculum) into filter-sterilized seawater supplied with N and P sources. PHBH films with dimensions of 1 cm×0.5 cm were placed into the seawater samples. After an incubation at 23°C for 3 d, PHBH films were taken from the culture medium and biofilm formation was assessed. In order to identify the bacterial species, the 16S rRNA gene was amplified by PCR using the method described above. After electrophoretic separation, amplified 16S rRNA was purified and sequenced using the BigDye Terminator v3.1 Cycle Sequencing Kit and 3500 Series Genetic Analyzer (Applied Biosystems, Tokyo, Japan). The closest type-strain 16S rRNA gene relatives of each clone sequence were assessed using the sequence match program of the Ribosomal Database Project (RDP-II) (4).

### Nucleotide sequence accession number

The nucleotide sequences reported in the present study were deposited in the DDBJ/ENA/GenBank database with the following accession numbers: LC221843–LC221845 (for 16S rRNA genes from strains E11, S23, and S35) and LC329349–LC330846 (for 16S rRNA clones from biofilm samples).

## Results

### Biofilm formation on PHBH film surfaces in seawater samples

**Fig. 1:**
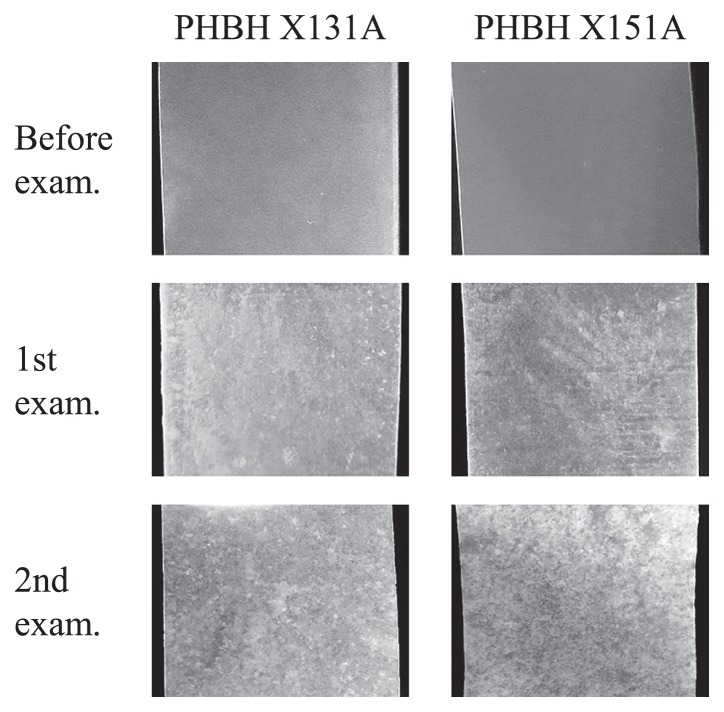
Biofilm formation on PHBH X131A and X151A film surfaces in seawater samples obtained at Takasago harbor on March 15 (1st exam) and April 19 (2nd exam), 2016.

In order to observe behaviors on the surfaces of biodegradable plastic films in seawater, six types of biodegradable plastic films, which were made from PLA, PBAT, PBS, PBSA, PCL, and PHBH, were incubated for 1 month in seawater samples obtained at Takasago harbor on March 15, 2016. Four plastic films: PLA, PBAT, PBS, and PBSA, did not show any changes on their surfaces. On the other hand, a certain amount of biofilms formed on the surfaces of the PHBH X131A and X151A films (Fig. 1). A small amount of biofilms was also observed on the surface of PCL films. Therefore, we examined the reproducibility of biofilm formation on PCL and PHBH films. These films were incubated in seawater samples obtained at Takasago harbor on April 19, 2016. After a 1-month incubation, a significant amount of biofilms was observed on two PHBH X131A films and three PHBH X151A films (Fig. 1). One each of the PHBH X131A and PCL films was partially degraded. PHBH and PCL films were also incubated in seawater samples obtained at Takasago harbor on June 8, 2016. All films tested were mostly or partially degraded after an incubation for 2 weeks. The temperature of seawater collected in June 2016 was higher than that collected in March and April 2016 (Table 2). This increase in seawater temperature was assumed to have caused a change in the bacterial community and increased the population of specific bacteria, which degrade PHBH plastics.

### Statistical analyses of clone libraries prepared from biofilms

In order to assess the composition of the bacterial community, clone libraries of the 16S rRNA genes were prepared and sequenced from the biofilms that formed on the surfaces of PHBH films. The statistical characteristics of these clone libraries are summarized in Table 3. A total of 89 to 96 clones were sequenced from each biofilm sample. Library coverage was considered to be experimentally high enough for most of the clone libraries (between 72.3% and 95.8%). Bacteria associated with mostly degraded PHBH films were shown to have the lowest diversities in the clone libraries. By analyzing the combined data set from the clone libraries for all samples, 255 OTUs were identified across all of the biofilms that formed on PHBH films.

### Phylogenetic analyses of biofilms on PHBH film surfaces

**Fig. 2:**
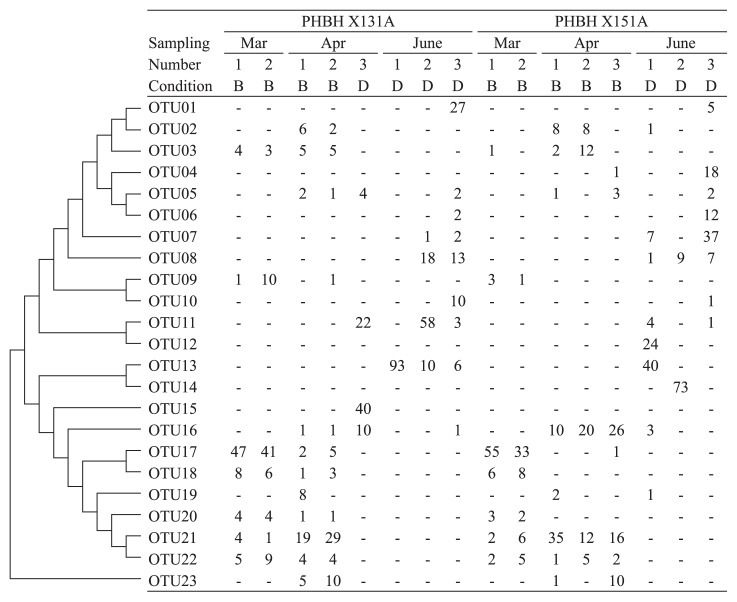
Phylogenetic distribution of OTUs based on 16S rRNA gene sequences of clone libraries. The dendrogram indicates phylogenetic relationships among the representative sequences of OTUs (defined by ≥97% identity). The table indicates the clone numbers belonging to each OTU in each library. The conditions of PHBH films were shown as B (forming biofilms) or D (mostly or partially degraded).

Analyses of phylogenetic compositions by the Classifier program of RDP-II revealed that the clone libraries were mainly dominated by 4 phyla, which contained the two major phyla *Proteobacteria* and *Bacteroidetes*. The most dominant phylum among all samples was *Proteobacteria*. Twenty-three OTUs, which contained more than 10 clones among all clones, were selected as the dominant OTUs in PHBH biofilms. These OTUs contained eight different families: *Rhodobacteraceae*, *Kordiimonadaceae*, *Erythrobacteraceae*, *Rhodospirillaceae*, *Oceanospirillaceae*, *Colwelliaceae*, *Alteromonadaceae*, and *Saprospiraceae* (Table 4). Details on the number of clones in each OTU are shown in Fig. 2. OTU23, which belongs to the phylum *Bacteroidetes*, was a minor member among all OTUs and only appeared in seawater samples collected in April 2016. On the other hand, another 22 OTUs, except for OTU23, which belong to the phylum *Proteobacteria*, were major members among all OTUs. In biofilms from unbroken PHBH films in seawater samples collected in March 2016, the most dominant OTU was OTU17, which showed high similarity with *Glaciecola lipolytica*. The other dominant OTUs were OTU18, OTU20, OTU21, and OTU22, and these were also assigned to the genus *Glaciecola*. In biofilms from PHBH films in seawater samples collected in April 2016, the dominant OTUs of unbroken PHBH films were assigned to the genera *Glaciecola* and *Colwellia*. On the other hand, the genus *Glaciecola* completely disappeared in biofilms from partially broken PHBH films. In seawater samples collected in June 2016, all PHBH films were mostly or partially broken within two weeks. The dominant OTUs were assigned to the families *Rhodobacteraceae*, *Rhodospirillaceae*, and *Oceanospirillaceae*, and the genus *Glaciecola* was hardly detected. These results assumed that the genus *Glaciecola* contributed to initial biofilm formation and the degradation of PHBH, but may have been removed by other bacteria that intercept PHBH degradation products after the partial fragmentation of PHBH films.

### Identification and characterization of PHBH-degrading strains

**Fig. 3:**
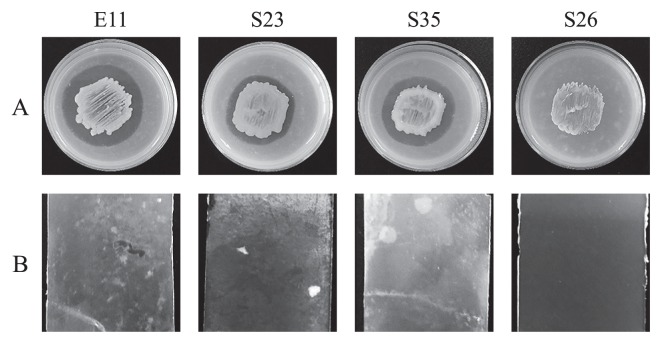
PHBH-degrading activity (A) and biofilm formation (B) of PHBH-degrading strains (E11, S23, and S35) and a negative control strain (S26). PHBH-degrading activity was detected on MB-PHBH agar plates after an incubation at 30°C for 3 d. The development of clear zones around the colonies was evaluated as the degradation of PHBH. Biofilm formation on the surface of PHBH X131A films was evaluated in filter-sterilized seawater after an incubation at 23°C for 3 d.

**Fig. 4:**
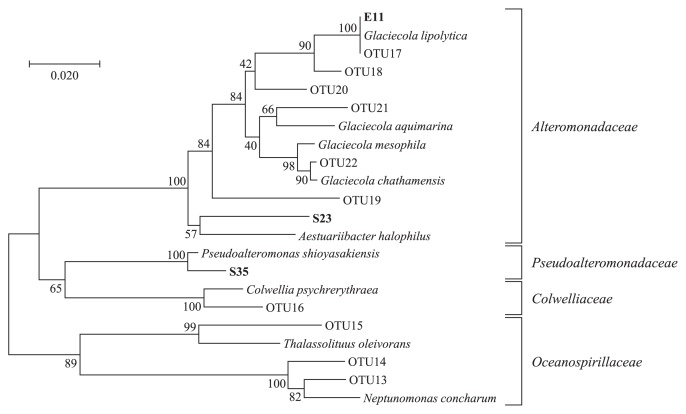
Phylogenetic tree of 16S rRNA gene sequences from PHBH-degrading isolates. The bacterial isolates in the present study were shown in bold style. The phylogenetic tree was constructed by the neighbor-joining method with the ClustalW program of MEGA. The percentage of replicate trees in which the associated taxa clustered together in the bootstrap test (1,000 replicates) are shown next to the branches. The scale bar represents 0.02 substitutions per nucleotide position.

Approximately 50 colonies isolated from the biofilm samples that formed on PHBH films were subjected to assessments of their PHBH-degrading activities on MB-PHBH plates. Three strains, E11, S23, and S35, showed the development of clear zones around the colonies after an incubation for 3 d (Fig. 3A). These strains were also evaluated for their biofilm formation abilities on PHBH films in seawater samples. After an incubation at 23°C for 3 d with shaking, three PHBH-degrading strains showed cell growth in seawater samples and formed significant biofilms on PHBH film surfaces (Fig. 3B). On the other hand, none of the strains showed cell growth or biofilm formation on any of the plastic films tested, except for PHBH (data not shown). In order to identify the bacterial species, we amplified and sequenced 16S rRNA genes. A phylogenetic analysis of the genes revealed that strains E11, S23, and S35 showed high similarity to *G. lipolytica* (>99% identity), *Aestuariibacter halophilus* (>97% identity), and *Pseudoalteromonas lipolytica* (>98% identity), respectively (Fig. 4). These results demonstrated that PHBH-degrading strains isolated from biofilms have specific PHBH-degrading activities.

## Discussion

PHBH is a PHA derivative that is expected to be used for various applications as a good biodegradable plastic. On the other hand, the mechanisms underlying the biodegradation of the processed goods of PHA such as plastic films have not been investigated in detail. In the present study, we focused on biofilm formation on PHBH film surfaces as a degrading agent. In the initial stages of the formation of biofilms, most of the clones in dominant OTUs were assigned to members of the family *Alteromonadaceae* or *Colwelliaceae*. A previous study identified two PHA-degrading bacterial strains isolated from a seawater environment as the genera *Shewanella* and *Marinobacter* of the family *Alteromonadaceae* (13, 26). In the present study, although the genus *Glaciecola* of the family *Alteromonadaceae* was mainly detected from biofilms on plastic surfaces, the genera *Shewanella* and *Marinobacter* were not contained in the dominant OTUs. The genera *Colwellia* and *Shewanella* have been reported as PHA-producing bacteria in the deep sea (19). The genus *Colwellia* of the family *Colwelliaceae* is a pre-dominant OTU in the biofilm on plastic surfaces. The genus *Colwellia* was assumed to have the ability to produce and degrade PHA, similar to the genus *Shewanella*.

In mostly or partially broken PHBH films, the dominant OTUs in the biofilms showed a prominent shift to the families *Oceanosprillanceae*, *Rhodobacteraceae*, and *Rhodospirillaceae*. In the family *Rhodospirillaceae*, *Rhodospirillum rubrum* has been reported as a bacterium that degrades PHA granules and expresses a putative intracellular PHA depolymerase (9). However, it currently remains unclear whether intracellular PHA-degrading enzymes have the ability to degrade extracellular PHA or its processed goods. In the family *Rhodobacteraceae*, a complete genome analysis of the PHA-producing bacterium *Yangia* sp. CCB-MM3 revealed the presence of gene homologues of PHA depolymerase (*phaZ*) (14). Intracellular PhaZ in PHA-producing bacteria is generally considered to be useful for catabolism for the reutilization of PHA in cells (11). Thus, these marine PHA-producing bacteria may only degrade the PHA monomer or oligomer attached to the periplasm or transported into the cell. If this is the case, the families *Rhodobacteraceae* and *Rhodospirillaceae* are assumed to have replaced PHBH-degrading *Alteromonadaceae* after the fragmentation of PHBH plastics into PHA monomers or oligomers. Based on these findings, we inferred that the biodegradation of PHBH films in seawater may be completed through two stages: (i) biofilm formation on film surfaces by PHBH-degrading bacteria, and (ii) transition to the bacterial community that intercepts PHBH degradation products.

In the present study, the plastic films tested, except for PHBH and PCL, were assumed to not be degraded or be degraded very slowly in seawater. Their biodegradability in fresh or seawater has also been reported. General biodegradable plastics have mainly been evaluated for their biodegradability in soil environments. In a previous study, the degradation and biofilm formation of eight different plastics were evaluated under uncontrolled composting conditions (18). Two out of five polyesters, PBS and PHA, showed low, but significant weight loss after a 450-d incubation in compost. In addition, the formation of a high cell-density biofilm was quantified on PBS and PHA. On the other hand, our results demonstrated that greater biofilm formation and degradation were observed on PHBH than on PBS. These results suggest that PHA derivatives are useful polyesters that may be easily biodegraded in a wide range of environments.

The biodegradation of PCL soaked in deep seawater has been evaluated in a previous study (23). Although PCL fibers show numerous heterogeneous pinholes and cracks after soaking in deep seawater, clear biofilm formation was not observed (23). Early biofilm formation on marine plastic debris made from polyethylene was observed (15); however, there was no indication that polyethylene-degrading microorganisms were present in these biofilms. Our results demonstrated that initial biofilm formation on PHBH film surfaces was important for the subsequent degradation stage, for example, by the mechanism used by biofilms to prevent the dispersion of the PHBH-degrading enzyme to the outside and increase the degradation efficiency of PHBH. Previous studies demonstrated that PHA-producing bacteria were present in marine environments and had potential as PHA-degrading bacteria. Therefore, PHBH combines the functionality of plastic for general use and its biodegradability better than other biodegradable plastics, and is expected to disappear due to biodegradation after entering the ocean. Further studies on the mechanisms responsible for biofilm formation and the degradation of PHBH films may contribute to molecular improvements in PHA derivatives and overcome microplastic issues.

## Figures and Tables

**Table 1:** Description of plastics used in the present study.

Polymer	Manufacturer	Product name	MW^a^
PLA	NatureWorks (USA)	Ingeo™ 10361D	160,000
PBAT	BASF (Germany)	EcoFlex^®^ C1200	120,000
PBS	Showa Highpolymer (Japan)	Bionolle™ 1020MD	150,000
PBSA	Showa Highpolymer (Japan)	Bionolle™ 3001MD	220,000
PCL	Perstorp (UK)	Capa™ 6500	150,000
PHBH (3HHx=6 mol%)	Kaneka (Japan)	KANEKA Biodegradable Polymer™ X131A	610,000
PHBH (3HHx=11 mol%)	Kaneka (Japan)	KANEKA Biodegradable Polymer™ X151A	550,000

a The weight average for molecular weight.

**Table 2:** Biofilm formation on plastic film surfaces in seawater samples.

Sampling date	Mar 15	Apr 19	June 8

Temp. (°C)	11	14	20
PLA	—a	N.T.^b^	N.T.
PBAT	—	—	N.T.
PBS	—	—	N.T.
PBSA	—	N.T.	N.T.
PCL	B^c^	D^d^	D
PHBH (X131A)	B, B	B, B, D	D, D, D
PHBH (X151A)	B, B	B, B, B	D, D, D

a —, nothing to change.

b N.T., not tested.

c B, forming biofilms.

d D, mostly or partially degraded.

**Table 3:** Characteristics of clone libraries from biofilms on PHBH films.

	PHBH X131A								PHBH X151A							
		
Sampling date	Mar 15		Apr 19			June 8			Mar 15		Apr 19			June 8		
						
Number of samples	1	2	1	2	3	1	2	3	1	2	1	2	3	1	2	3
						
Condition of films a,b	B	B	B	B	D	D	D	D	B	B	B	B	B	D	D	D
Number of sequences	90	94	94	94	94	96	96	95	92	89	92	96	94	93	94	95
Number of OTUs	17	22	39	36	17	4	12	28	22	30	33	35	31	18	11	15
Number of singletons	11	11	26	24	10	3	8	17	14	23	23	26	21	12	7	7
Library coverage (%)^c^	87.8	88.3	72.3	74.5	89.4	95.8	91.7	82.1	84.8	74.2	75.0	72.9	77.7	87.1	92.6	92.6

a B, forming biofilms.

b D, mostly or partially degraded.

c Library coverage (*C*_x_)=1–(*n*_x_/*N*), where *n*_x_ is the number of singletons that are encountered only once in a library and *N* is the total number of clones.

**Table 4:** Classification of OTUs from biofilms on PHBH film surfaces.

OTUs	Closest species	Family	Accession No.	Identity (%)
OTU01	*Primorskyibacter sedentarius*	*Rhodobacteraceae*	AB550558	96.4
OTU02	*Thalassobacter stenotrophicus*	*Rhodobacteraceae*	AJ631302	96.3
OTU03	*Sulfitobacter mediterraneus*	*Rhodobacteraceae*	Y17387	97.6
OTU04	*Phaeobacter inhibens*	*Rhodobacteraceae*	AY177712	96.8
OTU05	*Phaeobacter arcticus*	*Rhodobacteraceae*	DQ514304	99.4
OTU06	*Phaeobacter inhibens*	*Rhodobacteraceae*	AY177712	97.3
OTU07	*Tropicibacter naphthalenivorans*	*Rhodobacteraceae*	AB302370	97.6
OTU08	*Ruegeria mobilis*	*Rhodobacteraceae*	AB255401	99.7
OTU09	*Kordiimonas lacus*	*Kordiimonadaceae*	FJ847942	96.3
OTU10	*Altererythrobacter aestiaquae*	*Erythrobacteraceae*	KJ658262	89.9
OTU11	*Tistrella mobilis*	*Rhodospirillaceae*	AB071665	99.5
OTU12	*Insolitispirillum peregrinum*	*Rhodospirillaceae*	EF612768	97.6
OTU13	*Neptunomonas concharum*	*Oceanospirillaceae*	JF748732	96.8
OTU14	*Neptunomonas concharum*	*Oceanospirillaceae*	JF748732	96.6
OTU15	*Thalassolituus oleivorans*	*Oceanospirillaceae*	AJ431699	94.7
OTU16	*Colwellia psychrerythraea*	*Colwelliaceae*	AB011364	97.4
OTU17	*Glaciecola lipolytica*	*Alteromonadaceae*	EU183316	100
OTU18	*Glaciecola lipolytica*	*Alteromonadaceae*	EU183316	98.1
OTU19	*Aestuariibacter halophilus*	*Alteromonadaceae*	AY207503	94.1
OTU20	*Glaciecola mesophila*	*Alteromonadaceae*	AJ488501	97.6
OTU21	*Glaciecola arctica*	*Alteromonadaceae*	EU365479	97.6
OTU22	*Glaciecola chathamensis*	*Alteromonadaceae*	AB247623	99.5
OTU23	*Lewinella cohaerens*	*Saprospiraceae*	AF039292	99.4
